# Jiedu-Yizhi Formula Alleviates Neuroinflammation in AD Rats by Modulating the Gut Microbiota

**DOI:** 10.1155/2022/4023006

**Published:** 2022-07-31

**Authors:** Jiale Wang, Xiaoting Zhu, Yuhui Li, Wenping Guo, Mingquan Li

**Affiliations:** ^1^Traditional Chinese Medicine Department, Xijing Hospital of Air Force Military Medical University, Xi'an 710032, Shanxi, China; ^2^School of Chinese Medicine, Changchun University of Chinese Medicine, Changchun 130117, Jilin, China; ^3^Neurology Department, Changchun University of Chinese Medicine Third Affiliated Hospital, Changchun 130117, Jilin, China; ^4^School of Integrated Chinese and Western Medicine, Changchun University of Chinese Medicine, Changchun 130117, Jilin, China

## Abstract

**Background:**

The Jiedu-Yizhi formula (JDYZF) is a Chinese herbal prescription used to treat Alzheimer's disease (AD). It was previously confirmed that JDYZF can inhibit the expression of pyroptosis-related proteins in the hippocampus of AD rats and inhibit gut inflammation in AD rats. Therefore, it is hypothesized that JDYZF has a regulatory effect on the gut microbiota.

**Methods:**

In this study, an AD rat model was prepared by bilateral hippocampal injection of A*β*_25-35_ and AD rats received high, medium, and low doses of JDYZF orally for 8 weeks. The body weights of the AD rats were observed to assess the effect of JDYZF. The 16S rRNA sequencing technique was used to study the regulation of the gut microbiota by JDYZF in AD rats. Immunohistochemical staining was used to observe the expression levels of Caspase-1 and Caspase-11 in the hippocampus.

**Results:**

JDYZF reduced body weight in AD rats, and this effect may be related to JDYZF regulating body-weight-related gut microbes. The 16S rRNA analysis showed that JDYZF increased the diversity of the gut microbiota in AD rats. At the phylum level, JDYZF increased the abundances of *Bacteroidota* and *Actinobacteriota* and decreased the abundances of *Firmicutes*, *Campilobacterota,* and *Desulfobacterota*. At the genus level, the abundances of *Lactobacillus*, *Prevotella, Bacteroides*, *Christensenellaceae_R-7_group*, *Rikenellaceae_RC9_gut_group*, and *Blautia* were increased and the abundances of *Lachnospiraceae-NK4A136-group*, *Anaerobiospirillum*, *Turicibacter*, *Oscillibacter*, *Desulfovibrio*, *Helicobacter*, and *Intestinimonas* were decreased. At the species level, the abundances of *Lactobacillus johnsonii*, *Lactobacillus reuteri*, and *Lactobacillus faecis* were increased and the abundances of *Helicobacter rodentium* and *Ruminococcus_sp_N15.MGS-57* were decreased. Immunohistochemistry showed that JDYZF reduced the levels of Caspase-1- and Caspase-11-positive staining.

**Conclusion:**

JDYZF has a regulatory effect on the gut microbiota of AD rats, which may represent the basis for the anti-inflammatory effect of JDYZF.

## 1. Introduction

Alzheimer's disease (AD) can cause damage to cognitive domains such as memory, visuospatial processing, language, and personality and is the most common cause of dementia [1]. Worldwide, greater than 9.9 million new cases of AD are diagnosed each year, and 131.0 million people will suffer from AD by the middle of this century [2, 3]. In the past 20 years, the number of deaths due to AD has increased by 145%. In 2019 alone, approximately 1,300,000 people died of AD in the United States. AD has become the biggest killer of human life and health after cerebrovascular diseases and malignant tumours. The worldwide spread of the coronavirus disease 2019 (COVID-19) epidemic has increased the number of AD deaths [4]. The five drugs currently approved to treat AD improve the clinical symptoms of AD only for a period of time, and aducanumab, a new drug developed to treat the pathological *β*-amyloid (A*β*) deposition of AD typically observed in the clinic, has uncertain clinical efficacy [5, 6]. Therefore, it is particularly important to develop alternative medicines and therapies based on other theories of AD pathogenesis.

In long-term studies, A*β* overexpression and deposition are considered to be the culprit of AD. However, it cannot be ignored that A*β* is first and foremost an antimicrobial peptide, and its production is related to the response of nerve cells to infectious agents [7, 8]. For example, immunocompromised elderly individuals are susceptible to *Chlamydia pneumoniae* infection, and *Chlamydia pneumoniae* DNA is detected in 90% of AD patient's brain autopsy specimens. In addition, live *Chlamydia pneumoniae* has been isolated. How *Chlamydia* enters the brain is uncertain. Existing studies have confirmed that *Chlamydia pneumoniae* can infect peripheral nerves, such as the olfactory nerve and trigeminal nerve, and enter directly into the brain from the nasal cavity, and a large amount of A*β* can be induced and deposited within a short time after the emergence of *Chlamydia pneumoniae* in the brain. If peripheral infection persists, then this feedback will continue to occur, resulting in the overexpression and deposition of A*β* as well as the overactivation of microglia and the overproduction of inflammatory factors and reactive oxygen species [9]. This process subsequently creates an opportunity for the occurrence of AD. Central lesions resulting from gut microbiota disturbances follow a similar pattern. Short-chain fatty acids (SCFAs) are mainly produced by gut microbes and are involved in maintaining the integrity of the intestinal mucosal barrier and blood-brain barrier (BBB). Intestinal disorders during ageing can lead to a decrease in the level of SCFAs, resulting in a “leaky gut.” In this context, many immunogenic substances will then take the opportunity to cross the BBB and enter the brain, causing A*β* overexpression to induce neuronal death [10]. SCFAs are also involved in mediating the ability of microglia to respond to stimuli, and a reduction in SCFA levels will lead to the release of more proinflammatory factors and cytotoxins by microglia, which promotes further central inflammation [11]. In addition, experiments have confirmed that A*β* oligomers can migrate from the gut to the brain [12]. In addition, a large number of gut-resident bacteria and fungi can produce bacterial amyloids, although they do not share amino acid sequences with human A*β*. However, when the BBB is damaged, Toll-like receptors, RAGE receptors, and NOD-like receptors also recognize these bacterial amyloids when they enter the brain and induce microglia to release a large number of proinflammatory factors; thus, the brain is under the threat of chronic inflammation [13]. In addition, disturbances in the gut microbiota can lead to an increase in the production of lipopolysaccharide (LPS), and the involvement of LPS in destroying the intestinal mucosa worsens the “leaky gut” effect [14]. LPS can also circulate into the brain, stimulate the activation of microglia, and induce an inflammatory response. It has been observed that the levels of LPS in the hippocampus and cortex of AD patients are greater than those in healthy people [10]. Gut microbes are also producers of brain-derived neurotrophic factors and some neurotransmitters (such as acetylcholine, gamma-aminobutyric acid, and serotonin). Disturbed gut microbiota can lead to a decrease in the expression levels of these substances involved in normal cognitive function [15, 16]. These data on the disturbance of the gut microbiota seem to provide a perfect explanation of the mechanism underlying the pathogenesis of AD. Adjusting the disturbance of the gut microbiota can improve the cognitive function of AD patients in many ways, and current research has found that some substances and treatment methods have this effect. For example, mannan oligosaccharide (MOS) can reduce the abundance of *Helicobacter* in the gut of the 5XFAD transgenic AD mice, increase the abundance of *Lactobacillus* and some butyrate-producing microorganisms, and increase the expression of SCFAs. It may be related to the reduced A*β* deposition and downregulated inflammatory levels in the brain of AD mice [3]. L-Arginine and limonoids can increase the gut microbial diversity of App < NL-G-F > knock-in AD mice, thereby improving neuroinflammation and neurodegeneration [17]. Electroacupuncture in *Baihui* (GV20) and *Yintang* (GV29) of SAMP8 mice can increase the abundance of *Bacteroides* in their gut, reduce the abundance of *Clostridium*, and increase the ratio of *Bacteroides* to *Clostridium*. This effect is related to the inhibition of peripheral and central inflammation [2]. Is there a similar effect of Chinese herbal prescription?

The Jiedu-Yizhi formula (JDYZF) is a special prescription for AD created by Ren Jixue, a master of traditional Chinese medicine, based on the “marrow deficiency and toxin damage” theory. In a previous study of AD rats, we found that JDYZF improved the cognitive impairment of AD rats induced by A*β*_25–35_, reduced the expression levels of A*β* and pyroptosis-related proteins in the hippocampus of AD rats, and downregulated the expression levels of A*β*_1–42_, interleukin (IL)-1*β*, and IL-18 in the hippocampus and cortex, thereby reducing neuroinflammation. In addition, JDYZF downregulates the expression levels of A*β*_1–42_, IL-1*β*, and IL-18 in the gut [18], so we hypothesize that JDYZF may regulate the gut microbiota of AD rats. Based on the above observations, in this study, we continued to use the AD rat model created by bilateral hippocampal injection of A*β*_25–35_ as the observation object to explore the effect of JDYZF on the gut microbiota of AD rats and analyse whether it is the basis for its neuroprotective effect.

## 2. Materials and Methods

### 2.1. Animals

Yisi Experimental Animal Technology Co., Ltd. (Changchun, China), provided adult male SD rats (weight: 200–220 g). The animals were allowed to drink water and eat freely during the feeding and research period. The ambient temperature was 25 ± 3°C, the relative humidity was 55 ± 5%, and a 12-hour light-dark cycle was followed. The animal experiment procedure was approved by the Animal Ethics Committee of Changchun University of Chinese Medicine (no. 2021207).

### 2.2. Preparation of the A*β*_25–35_ Oligomers and Modelling

We dissolved 1mg A*β*_25–35_ (A4559, Sigma) dry powder in 500 ul of 0.9% normal saline to prepare a 2 ug/ul solution and incubated for 7 days in a 37°C incubator after sonication, and the incubated A*β*_25–35_ oligomers were flocculent and stored in a refrigerator at 4°C for later use. Sodium pentobarbital was selected as an anaesthetic for rats, and we used a brain stereotaxic apparatus to fix and mark the coordinates of rats' heads. We prepared the head skin of the rats and drilled two holes (coordinates: 3 mm below the bregma and 2 mm on both sides of the midline) in the skull with a dental drill. We absorbed 5 ul of A*β*_25–35_ solution with a microsyringe and fixed it on the injection frame of the brain stereotaxic apparatus. We manipulated the instrument to lower the injection needle and probe into the hole, pierced the subdural by 2.6 mm, slowly injected the solution within 5 minutes, stopped for 15 minutes after the injection, and slowly withdrew the needle within 5 minutes. The skull hole was closed with paraffin, and the injection procedure was the same on the other side. After 7 days of injection, surviving rats can become AD models [19, 20].

### 2.3. Preparation of the JDYZF Decoction

JDYZF includes *Coptis chinensis Franch (Ranunculaceae)*, *wine-treated Rheum palmatum* L *(Polygonaceae)*, *Ligusticum striatum DC (Apiaceae)*, Geosaurus *(Pheretima aspergillum), Carapax Testudinis paste (Chinemys reevesii (Gray))*, *Cornus officinalis Siebold & Zucc (Cornaceae)*, and *Alpinia oxyphylla Miq (Zingiberaceae)*. According to the ratio of 1 : 1:1 : 1:1 : 1:2, the purchased herbal medicine was required for gavage for eight weeks. After two decoctions, the obtained liquid was concentrated to 1.0 g/mL and stored at −20°C.

### 2.4. Animal Grouping and Treatment

SD rats in the same batch were taken as the control group (CG), and the model rats were randomly divided into the model group (MG), donepezil hydrochloride group (PG), and JDYZF low-dose group (JDYZ.L), middle-dose group (JDYZ.M), and high-dose group (JDYZ.H); the number of rats were 9, 10, 10, 11, 11, and 11 in each group. The CG group and the MG group were given normal saline at a dose of 1 ml/100 g; the PG group was given the donepezil hydrochloride (Eisai, H20050978) suspension at a dose of 0.9 mg/kg, and the three groups of JDYZ.L, JDYZ.M, and JDYZ.H were given the drug solution of JDYZF by gavage at the doses of 3.6 g/kg, 7.2 g/kg, and 14.4 g/kg, respectively. All rats were administered the doses intragastrically once daily for 8 weeks.

### 2.5. Data and Sample Collection

The body weight of the rats was measured once after purchase and once every other week during the gavage. Eight weeks after gavage, the rat faeces were collected with disposable sterile medical forceps, put into cryopreservation tubes, and sealed with parafilm. All sampling times were kept within 2 hours and then stored in a −80°C refrigerator to avoid repeated freezing and thawing. Anaesthetized rats with sodium pentobarbital and hippocampal tissue were taken quickly, put into liquid nitrogen, and then stored in a −80°C refrigerator.

### 2.6. Faecal 16S rRNA Sequencing

The total genomic DNA of the sample was extracted using the cetyltrimethylammonium bromide (CTAB) method, and the DNA was diluted to 1 ng/*μ*L with sterile water. The V3-V4 region of the 16S rRNA gene was amplified using barcoded specific primers (341F:CCTAYGGGRBGCASCAG, 806R:GGACTACNNGGGTATCTAAT). All PCR experiments were performed using 15 *μ*L of Phusion® High-Fidelity PCR Master Mix (New England Biolabs). The same volume of 1X loading buffer (containing SYB green) was mixed with the PCR product and detected by electrophoresis on a 2% agarose gel. PCR products were mixed in equidensity ratios, and the mixed PCR products were purified with a Qiagen Gel Extraction Kit (Qiagen, Germany). After generating sequencing libraries using the TruSeq® DNA PCR-Free Sample Preparation Kit (Illumina, USA), the library quality was assessed with the Qubit@ 2.0 Fluorometer (Thermo Scientific) and Agilent Bioanalyzer 2100 system and finally sequenced with the Illumina NovaSeq platform to generate 250-bp paired-end reads.

### 2.7. Gut Microbiota Analysis

After subtracting the barcodes and primer sequences of paired-end reads, the FLASH tool was used to splice the reads of each sample [21], and after quality filtering was performed according to the quality control process of Quantitative Insights into Microbial Ecology (QIIME) [22], the UCHIME algorithm was used to detect and remove chimaeras and to finally obtain the effective tags [23, 24]. Effective tags were clustered into operational taxonomic units (OTUs) with 97% consistency using the UPARSLE algorithm [25]. Species annotation analysis was performed with the SILVA database to obtain taxonomic information [26], and at each taxonomic level, phylum, class, order, family, genus, and species were used to assess the community composition of each sample. QIIME software and R software were used to calculate and plot various conventional alpha-diversity and beta-diversity values. The differences between groups in the diversity index were analysed using Tukey's test and the Wilcoxon test of the R software agricolae package. Metastats analysis was performed at each classification level using R software, and the *p* value was corrected to obtain the *q* value. The linear discriminant analysis effect size (LEfSe) method was performed using LEfSe software, and the default setting of the linear discriminant analysis (LDA) score filter value was 4.

### 2.8. Immunohistochemistry

4um sections of the hippocampus were made, and immunohistochemical staining was performed according to the conventional method [27]. The primary antibodies were Caspase-1 (1 : 100, Novus, USA) and Caspase-11 (1 : 100, Novus, USA). Cytation 5 (BioTek, USA) image reader was used to observe and take pictures, and Image *J* was used to analyse the average optical density (AOD).

### 2.9. Statistical Analysis

The body weight of rats and immunohistochemical data were presented as the mean ± standard deviation, and one-way analysis of variance (one-way ANOVA) with SPSS 25 software was used to compare the differences among multiple groups. Analysis of gut microbiome data was included above. *p* value and *q* value of < 0.05 were considered statistically significant, and *p* value and *q* value of < 0.01 were considered highly statistically significant.

## 3. Results

### 3.1. The  Effect of JDYZF on the Body Weight of AD Rats

**Figure 1:**
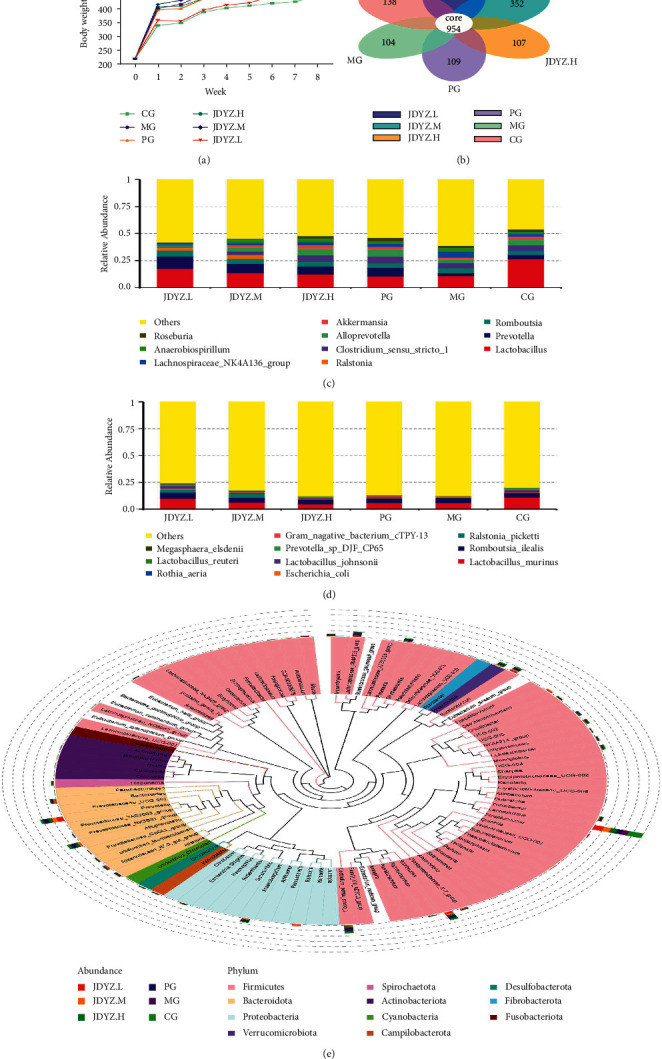
(a) Weight growth degree and trend of rats in each group; (b) the number of common and unique OTUs in each group and petal diagram; (c) the top 10 species in abundance at the genus level; (d) the top 10 species at the species level; (e) phylogenetic relationships of species at the genus level.

By weighing the rats, it was found that the body weight of the rats was maintained at the same level as that at the time of purchase. From the time of purchase to the end of the first week of gavage, the rats underwent adaptive feeding, premodelling, modelling, and gavage for 7 days for a total of 32 days. The body weight of the rats changed significantly. The body weight of the CG group was the lowest, and the body weight of the MG, PG, JDYZ.H, and JDYZ.M groups was significantly greater than that of the CG group (*p*=0.002, *p*=0.004, *p*=0.001, and *p*=0.000). No significant differences were noted among the other four groups. The body weight of JDYZ.L group was less than that of the MG group (*p*=0.02) and was not significantly different from that of the CG group. After 8 weeks of gavage, the body weight of the CG group was the lowest, the body weights of the MG and JDYZ.H groups were higher than those of the CG group (*p*=0.005, *p*=0.01), and the body weights of the PG, JDYZ.M, and JDYZ.L groups were higher than those of the CG group, but the difference was not significant (Table 1 and Figure 1(a)).

### 3.2. The Effects of JDYZF on 16S rRNA in the Gut Microbiota in AD Rats

A total of 62 rat faecal samples were sequenced to obtain raw paired-end reads (Raw PE), splicing raw PE, filtering low-quality and short-length sequences, and filtering chimaeras to obtain effective tags for analysis. Among them, the average numbers of raw PE and effective tags in the CG group were 96,538 and 60,523; 94,723 and 58,760 in the MG group; 98,760 and 60,906 in the PG group; 97,921 and 59,599 in the JDYZ.H group; 100,802 and 62,441 in the JDYZ.M group; and 104206 and 64347 in the JDYZ.L group, respectively. The average length (AvgLen) of all effective tags was 415 bp (Table 2).

### 3.3. OTU Analysis of the Gut Microbiota in AD Rats

According to the OTUs obtained by clustering, the common and unique OTUs among different groups were analysed and a petal diagram was drawn after normalization. The results revealed 954 common OTUs in all groups, 138 unique OTUs in the CG group, 104 unique OTUs in the MG group, 109 in the PG group, 107 in the JDYZ.H group, 352 in the JDYZ.M group, and 510 in the JDYZ.L group (Figure 1(b)). According to the species annotation results, the top 10 species in each group at the genus and species taxonomic levels were selected to generate a column accumulation chart of relative abundance. The results showed that *g_lactobacillus* and *g_prevotella* were more abundant in each group at the genus level (Figure 1(c)) and *s_lactobacillus-murinus*, *s_ralstonia-pickettii*, and *s_lactobacillus-johnsonii* were more abundant at the species level (Figure 1(d)). In addition, proportional differences were noted. The genus-level species evolutionary relationship diagram shows the differences in each group of genus-level species in another form and clarifies the evolutionary relationship of genus-level species. Here, the colours of branches and sectors indicate their corresponding phyla, and the stacked column outside the fan ring shows the abundance information of the genus in different groups (Figure 1(e)).

### 3.4. Alpha-Diversity Analysis

**Figure 2:**
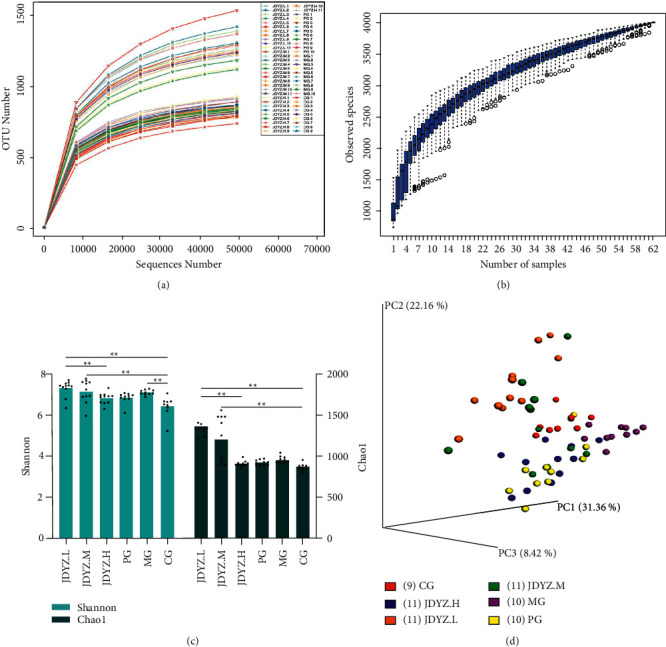
(a) Rarefaction curve; (b) species accumulation boxplot; (c) the boxplot of the Shannon index difference between groups; (d) three-dimensional PCoA map based on weighted UniFrac distance.

As shown in Figure 2(a), the rarefaction curve tends to be flat. This finding indicates that as the amount of sequencing data increases, the number of new OTUs will not increase significantly. As shown in Figure 2(b), in the species accumulation boxplot, with the increase in the number of samples, the increase in species diversity also tended to be moderate. These two figures show that the amount of sequencing data and the number of samples in this study are basically reasonable. The differences in the alpha-diversity index of the samples in each group were analysed. The Shannon index of the MG group was greater than that of the CG group (*p*=0.0002), while those of the PG and JDYZ.H groups were higher than that of the CG group and lower than that of the MG group, but the difference was not statistically significant; those of the JDYZ.M and JDYZ.L groups were significantly higher than those of the CG group (*p*=0.0002, *p* < 0.0001), and that of the JDYZ.L group was higher than that of the MG group, but there was no statistical significance. Regarding the Chao1 index, compared with the CG group, the MG group had no statistical difference. Compared with the MG group, the index of JDYZ.M and JDYZ.L groups increased, but there was no statistical difference and the index of both groups was significantly higher than of the CG group (*p* < 0.0001 and *p* < 0.0001) (Figure 2(c)).

### 3.5. Beta-Diversity Analysis

Principal component analysis (PCoA) was performed based on the weighted UniFrac distance, and three principal coordinates, PC1, PC2, and PC3, that describe 31.36%, 22.16%, and 8.42% of the total variation in the original sample, respectively, were selected to generate a three-dimensional PCoA map. As shown in Figure 2(d), samples from each group formed intragroup aggregates and had clear boundaries with other groups, indicating that the structure of the gut microbiota in each group had changed.

### 3.6. Species Analysis of Differences between Groups

**Figure 3:**
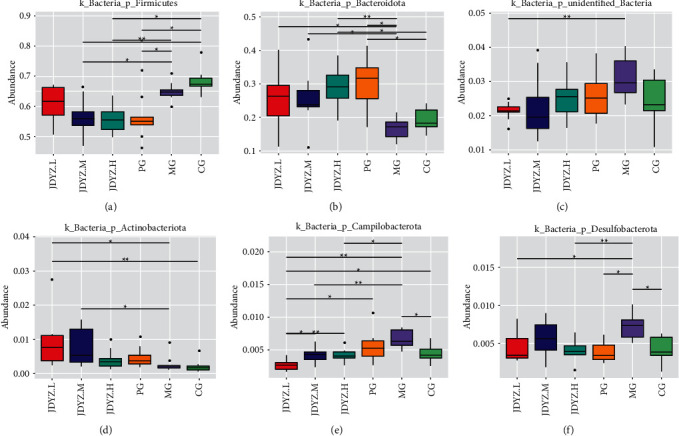
Phyla with significant differences in abundance at the phylum level in each group of rats. (a) *p_Firmicutes*; (b) *p_Bacteroidota*; (c) *p_Unidentified_Bacteria*; (d) *p_Actinobacteriota*; (e) *p_Campilobacterota*; (f) *p_Desulfobacterota*. Comparison between the groups: ^*∗*^*q* < 0.05 and ^*∗∗*^*q* < 0.01.

Starting from the species abundance table at the phylum, genus, and species classification levels, the MetaStat method was used to screen for species with significant differences in each group and the abundance boxplot was drawn. At the phylum level, the abundance of *p_Firmicutes* in the MG group was lower than that in the CG group, but there was no statistical difference, and the PG, JDYZ.H, and JDYZ.M groups had a lower abundance of *p_Firmicutes* than the MG group (*q* = 0.011, *q* = 0.008, *q* = 0.011), while that of the JDYZ.L group had no significant difference from those of the CG and MG groups (Figure 3(a)). Regarding *p_Bacteroidota* abundance, that of the MG group was lower than that of the CG group and those of the PG and JDYZ.H, JDYZ.M, and JDYZ.L groups were higher than those of the MG group (*q* = 0.011, *q* = 0.008, *q* = 0.011, *q* = 0.019) (Figure 3(b)). The abundance of *p_Unidentified_Bacteria*, *p_Campilobacterota*, and *p_Desulfobacterota* was increased in the MG group compared with the CG group. The levels in all four treatment groups were lower than those of the MG group, among which the JDYZ.L group had the most significant decrease (*q* = 0.003, *q* = 0.003, *q* = 0.015) (Figures 3(c), 3(e), and 3(f)). *P_Actinobacteriota* abundance in the MG group was not significantly different from that of the CG group; however, increased levels were noted in the JDYZ.L group compared with the MG group (*q* = 0.012) (Figure 3(d)).

**Figure 4:**
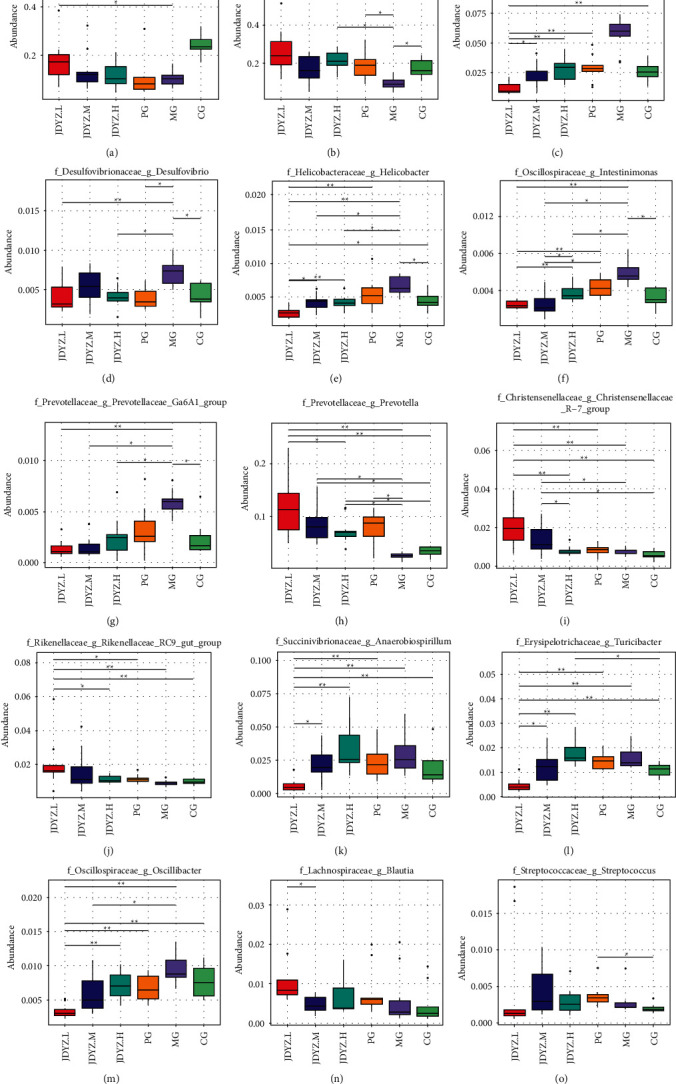
Genera with significant differences in abundance at the genus level in each group of rats. (a) *g_lactobacillus*; (b) *g_bacteroides*; (c) *g_Lachnospiraceae-NK4A136-group*; (d) *g_Desulfovibrio*; (e) *g_Helicobacter*; (f) *g_Intestinimonas*; (g) *g_Prevotellaceae_Ga6A1_group*; (h) *g_prevotella*; (i) *g_Christensenellaceae_R-7_group*; (j) *g_Rikenellaceae_RC9_gut_group*; (k) *g_Anaerobiospirillum*; (l) *g_Turicibacter*; (m) *g_Oscillibacter*; (n) *g_Blautia*; (o) *g_Streptococcus*. Comparison between the groups: ^*∗*^*q* < 0.05 and ^*∗∗*^*q* < 0.01.

At the genus level, the abundances of *g_lactobacillus* and *g_bacteroides* in the MG group were lower than those in the CG group (*q* = 0.026, *q* = 0.026), whereas the JDYZF groups had higher abundances than the MG group with the levels noted in the JDYZ.L group being the most significant (*q* = 0.042, *q* = 0.0038) (Figures 4(a) and 4(b)). Levels of *g_Lachnospiraceae-NK4A136-group*, *g_Desulfovibrio*, *g_Helicobacter*, *g_Intestinimonas*, and *g_Prevotellaceae_Ga6A1_group* were increased in the MG group compared with the CG group (*q* = 0.026, *q* = 0.047, *q* = 0.047, *q* = 0.026, *q* = 0.037). The JDYZF group had lower abundances of these genera than those of the MG group, with the JDYZ.L group showing the most significant difference (*q* = 0.0038, *q* = 0.0067, *q* = 0.0038, *q* = 0.0038, *q* = 0.0038) (Figures 4(c)–4(g)). Levels of *g_prevotella*, *g_Christensenellaceae_R-7_group*, and *g_Rikenellaceae_RC9_gut_group* were not significantly different in the MG group compared with the CG group. The JDYZF groups had higher abundances of these genera than the MG group, and the levels in JDYZ.L group were the most significant (*q* = 0.0038, *q* = 0.0038, *q* = 0.0038) (Figures 4(h)–4(j)). Levels of *g_Anaerobiospirillum*, *g_Turicibacter*, and *g_Oscillibacter* abundances were not significantly different in the MG group compared with the CG group. The JDYZF groups had a lower abundance of these genera than the MG group, and levels noted in the JDYZ.L group were the most significant (*q* = 0.0038, *q* = 0.0038, *q* = 0.0038) (Figures 4(k)–4(m)). Regarding g*_Blautia* abundance, no significant differences were noted between the MG group and the CG group. The JDYZ.L group had a higher abundance of this genus than the MG group, but the difference was not significant (Figure 4(n)). Regarding *g_Streptococcus* abundance, there was no significant difference between the MG group and the CG group. The JDYZ.L group had a lower abundance of this genus than the MG group, but the difference was not significant (Figure 4(o)).

**Figure 5:**
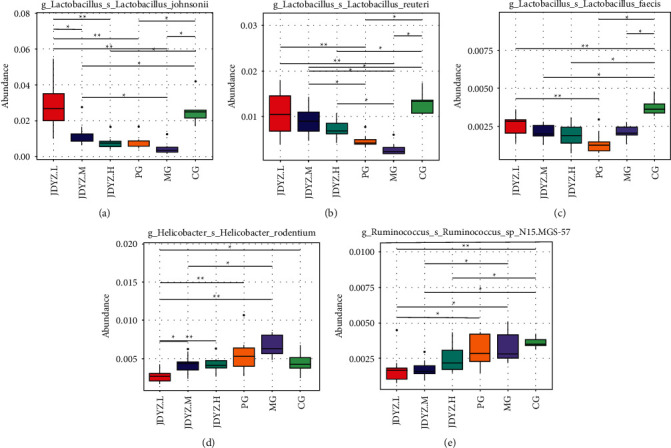
Species with significant differences in abundance at the species level in each group of rats. (a) *s_Lactobacillus_johnsonii*; (b) *s_Lactobacillus_reuteri*; (c) *s_Lactobacillus_faecis*; (d) *s_Helicobacter_rodentium*; (e) *s_Ruminococcus_sp_N15.MGS-57*. Comparison between the groups: ^*∗*^*q* < 0.05 and ^*∗∗*^*q* < 0.01.

At the species level, *s_Lactobacillus_johnsonii* and *s_Lactobacillus_reuteri* abundances were lower in the MG group than in the CG group (*q* = 0.041, *q* = 0.041). The JDYZF groups had higher abundances of these species than the MG group, and the levels in the JDYZ.L group were the most significant (*q* = 0.0042, *q* = 0.0042) (Figures 5(a) and 5(b)). The abundance of *s_Lactobacillus faecis* in the MG group was lower than that in the CG group (*q* = 0.041). The JDYZ.L group had a higher abundance of this genus than the MG group, but the difference was not significant (Figure 5(c)). *S_Helicobacter_rodentium* and *s_Ruminococcus_sp_N15. MGS-57* abundances were higher in the MG group than in the CG group, but the difference was not significant. The JDYZF groups had lower abundances of these two genera than the MG group, and the levels noted in the JDYZ.L group were the most significant (*q* = 0.0042, *q* = 0.011) (Figures 5(d) and 5(e)).

**Figure 6:**
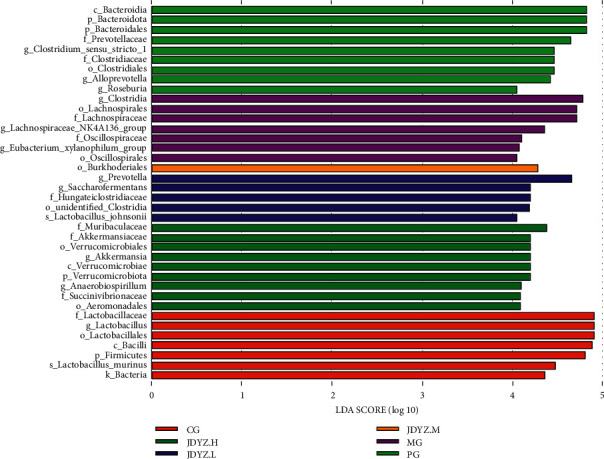
Histogram of the distribution of LDA values. The LDA score greater than 4 was regarded as the biomarker with the statistical difference between groups. The length of the histogram represents the effect size of the different species.

Using the LEfSe tool to compare multiple groups, the results showed that *f_Lactobacillaceae*, *g_Lactobacillus*, etc., were the dominant bacteria in the CG group; *c_Clostridia*, *o_Lachnospirales*, etc., were the dominant bacteria in the MG group; *c_Bacteroidia*, *p_Bacteroidota*, etc., were the dominant bacteria in the PG group; *f_Muribaculaceae*, *f_Akkermansiaceae*, etc., were the dominant bacteria in the JDYZ.H group; *o_Burkholderiales* was the dominant bacteria in JDYZ.M group; and *g_Prevotella*, *g_Saccharofermentans*, *f_Hungateiclostridiaceae*, *o_unidentified_Clostridia*, and *s_Lactobacillus_johnsonii* were the dominant bacteria in the JDYZ.L group (Figure 6).

### 3.7. JDYZF Reduces the Positive Reaction Degree of Caspase-1 and Caspase-11 in the Hippocampus of AD Rats

**Figure 7:**
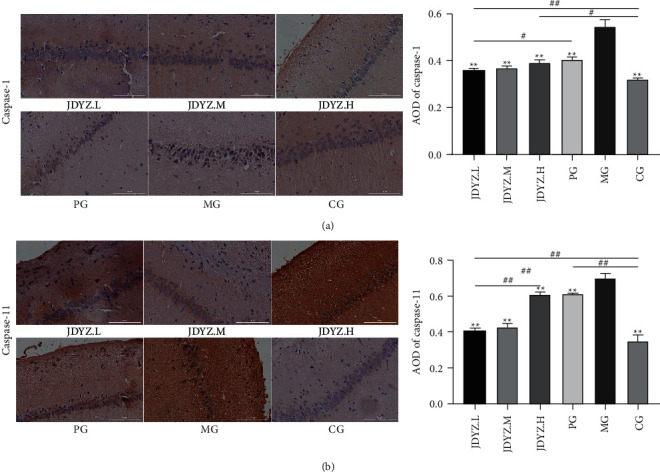
(a) Caspase-1 immunohistochemical staining and AOD levels in the hippocampus of rats in each group; (b) Caspase-11 immunohistochemical staining and AOD levels. ^*∗∗*^*p* < 0.01, other groups are compared with the model group. ^#^*p* < 0.05 and ^##^*p* < 0.01, other groups are compared in pairs except for the model group.

An immunohistochemical method was used to assess the positivity for Caspase-1 and Caspase-11 of the rat hippocampal tissue from each group. The results showed that the average optical density (AOD) of Caspase-1 and Caspase-11 hippocampal slices in the MG group was higher than that in the CG group (*p* < 0.01, *p* < 0.01). The AOD of the PG, JDYZ.H, JDYZ.M, and JDYZ.L groups was lower than that of the MG group, and the values noted in the JDYZ.L group were the most significant (*p* < 0.01, *p* < 0.01) (Figures 7(a) and 7(b)).

## 4. Discussion

Significantly different gut microbiota were observed in AD patients and normal subjects. It is difficult to say whether the impairment of central nervous system function caused by AD leads to the lack of brain regulation of the enteric nervous system first, which leads to changes in the gut microbial environment, or whether it is the imbalance of the gut microbiota caused by multiple factors that induces AD first. However, it is an established fact that the brain and gut microbiota significantly influence each other. A*β*_25–35_ hippocampal injection can induce AD and lead to impaired cognitive function in rats. In addition, this study found that hippocampal A*β*_25–35_ injection caused the species abundance and microbial structure of rats to deviate from those of normal rats; this deviation may be involved in the occurrence of cognitive impairment. JDYZF improved the cognitive impairment of AD rats and caused changes in the gut microbiota. Therefore, it is hypothesized that the regulation of the gut microbiota by JDYZF is closely related to the improvement of cognitive ability.

At the phylum level, JDYZF amplifies the decrease in the abundance of *p_Firmicutes* and simultaneously increases the abundance of *p_Bacteroidota*. Previous studies supported the result that the abundance of *p_Firmicutes* decreased and *p_Bacteroidota* increased in AD patients [28]. However, other studies have been published supporting a positive correlation between increased cognitive impairment and increased abundance of *p_Firmicutes* and decreased abundance of *p_Bacteroidota* [29, 30]. In addition, elevated levels of *p_Firmicutes* are responsible for obesity and higher TNF-*α* levels in the population [31, 32]. *P_Bacteroidota*, which has always been regarded as an opportunistic pathogen in AD, has increased abundance when probiotics are used to treat AD with curative effects [33–35]. Therefore, it is impossible to judge whether the changes in the abundance of *p_Firmicutes* and *p_Bacteroidota* have positive or negative effects on AD from a single perspective.

From the perspective of Chinese medicine, the main pathogenesis of AD is “marrow deficiency and toxin damage,” toxins mainly are composed of phlegm and blood stasis, and obesity is synonymous with the accumulation of phlegm and blood stasis in the body. *p_Firmicutes* and *p_Bacteroidota* abundances were positively and negatively correlated with obesity, respectively [36]. JDYZF reduces the abundance of *p_Firmicutes* and increases the abundance of *p_Bacteroidota*, which inhibits the occurrence of obesity, reduces the chance of phlegm and blood stasis, and is beneficial to AD brains attacked by toxins. In addition, rats treated with low-dose JDYZF had a higher abundance of *p_Actinobacteriota* than that of untreated AD rats, and these microbes can produce a large amount of SCFAs to repair the intestinal barrier and reduce the occurrence of “leaky gut” [37]. Its subordinate *S_Bifidobacterium* can reduce the amount of LPS transferred from the intestine to the serum, help reduce the occurrence of systemic chronic inflammation [38], and reduce the production of inflammatory cytokines to downregulate inflammation [39], which is undoubtedly beneficial to improve AD. JDYZF also reduced the abundance of *p_Campilobacterota*, a phylum that causes bacterial diarrhoea and multiple systemic infections [40]. For AD, a disease with background inflammation, it is a risk factor and the inhibition of *p_Campilobacterota* by JDYZF has a positive effect.

At the genus level, JDYZF increased the abundances of *g_lactobacillus*, *g_prevotella*, *g_bacteroides*, *g_Christensenellaceae_R-7_group*, *g_Rikenellaceae_RC9_gut_group*, and *g_Blautia*. Among them, *g_Lactobacillus* is important for maintaining gut homeostasis. Studies have shown that the abundance of *Lactobacillus* in the gut of AD patients is reduced [41] and AD can be improved by supplementation with *Lactobacillus* [42]. This finding may be related to the fact that *Lactobacillus* can reduce the deposition of A*β* in the brain, upregulate the levels of acetylcholine and brain-derived neurotrophic factor (BDNF), and inhibit the inflammatory response in the brain [43, 44]. *G_prevotella* is an important producer of SCFAs involved in maintaining the integrity of the gut mucosal barrier, and its abundance is positively correlated with the level of BDNF in the blood, which has important effects on learning and memory [45]. *G_bacteroides* is a potential probiotic that plays an important role in maintaining gut ecological balance, regulating lymphocyte and cytokine expression, controlling metabolism, and reducing inflammation and is closely related to neurodevelopment [46, 47]. *g_Christensenellaceae*, *g_Rikenellaceae*, and *g_Blautia* abundances were found to be negatively correlated with obesity and visceral fat content. In this study, these microbes may be involved in the effect of weight change in rats. In addition, they can antagonize gut inflammation, are markers of gut health, and are potential beneficial bacteria for AD with brain-gut interactions. More interestingly, *G_Blautia* can downregulate fasting blood glucose and glycosylated haemoglobin levels and participate in maintaining blood glucose homeostasis, and it is well known that diabetic glucose metabolism disorder plays an important role in the pathogenesis of AD and persists during AD. The subsequent effects of upregulation of *g_Blautia* abundance could be enormous. Berberine intake can specifically increase the abundance of *g_Blautia*. In addition, the main component of *Coptis*, one of the main medicines of JDYZF, is berberine, which explains why JDYZF upregulates the abundance of *g_Blautia* [48–51]. On the other hand, JDYZF also reduced the abundances of *g_Lachnospiraceae-NK4A136-group*, *g_Anaerobiospirillum*, *g_Turicibacter*, *g_Oscillibacter*, *g_Desulfovibrio*, *g_Helicobacter*, *g_Intestinimonas*, *g_Streptococcus*, and other genera. Among them, the biological role of the *g_Lachnospiraceae-NK4A136 group* is complex. This group is not only a producer of SCFAs but also a mediator of obesity and diabetes and an activator of inflammation [52]. Antiobesity treatment with resveratrol reduced the abundance of the *Lachnospiraceae-NK4A136 group*, and this process was accompanied by a reduction in gut inflammation and the repair of the gut barrier [53, 54]. The downregulation of this genus by JDYZF may also have a similar effect. *G_Anaerobiospirillum* is a potentially pathogenic bacterium that causes bacteraemia and gut infections with high mortality [55]. *G_Turicibacter* and *G_Oscillibacter* are closely related to immune diseases and gut inflammation [56, 57], and their abundances are elevated in patients with depression [58]. These bacteria may play more negative roles in gut-brain interaction processes. *G_Desulfovibrio* abundance is elevated in Parkinson's disease patients, and it produces a large amount of LPS to induce oligomerization and aggregation of *α*-synuclein [59]. Similarly, LPS-induced inflammation is also responsible for the oligomerization and aggregation of A*β*. In addition, *g_Desulfovibrio* abundance is elevated in anxiety and depression patients, which may be related to its role as a proinflammatory species that induces peripheral inflammation to cause stress to the brain [60]. Of course, it can also be considered that the peripheral inflammation induced by *g_Desulfovibrio* is one of the reasons for the background inflammation in AD. *G_Helicobacter* is the most studied pathogenic bacteria and is significantly related to gastrointestinal inflammation and ulcers. In research on the correlation between *g_Helicobacter* and AD, it was found that intraperitoneal injection of *Helicobacter* can directly induce the overexpression of A*β* and hyperphosphorylation of tau in the brains of rats; furthermore, the LPS produced by *g_Helicobacter* has been associated with autoimmune complications of neuropathy [61]. Studies of cocultures of human gastric cells MNK-28 with Hp peptide found that genes with AD characteristics, such as *APP*, *APOE*, *PSEN1*, and *PSEN2*, were activated in the cells. More than 70 genes were activated, of which 30 belong to the inflammatory pathway [62]. In addition, *Helicobacter* induced the overexpression of TNF-*α* and IL-1*β* in the periphery and mediated the damage to gastrointestinal epithelial cells and the BBB. Thus, TNF-*α* and IL-1*β* easily enter the brain [61]. The contribution of *g_Helicobacter* to AD was significant, and a low dose of JDYZF had a strong downregulating effect on this response. *G_Intestinimonas* is another genus that is positively associated with obesity [63], but it is controversial in terms of whether it promotes or inhibits inflammation. For example, an increase in the abundance of *g_Intestinimonas* was observed in Huntington's disease, and it was positively correlated with IL-4 levels in the blood, demonstrating anti-inflammatory activity [64]. Some studies have also suggested that it has proinflammatory properties, which are manifested by inducing immune cells to produce excessive inflammatory mediators [65]. The colonization of *Streptococcus sativa* is positively correlated with obesity, diabetes, and diabetes-associated infections and can also cause primary multisystem inflammation, such as enteritis, pneumonia, and meningitis. *Streptococcus* is one of the most invasive bacterial genera in humans [66–68]. In addition, *Streptococcus* can enter the brain through the gut barrier and blood, leading to activation of microglia and overexpression of ROS and A*β* [69].

At the species level, JDYZF increased the abundances of *s_Lactobacillus_johnsonii*, *s_Lactobacillus_reuteri*, and *s_Lactobacillus_faecis* but decreased the abundances of *s_Helicobacter_rodentium* and *s_Ruminococcus_sp_N15.MGS-57*. *S_Lactobacillus_johnsonii* is a high-level producer of acetic acid, butyric acid, and lactic acid, which can slow down the consumption of SCFAs during infection by pathogenic bacteria [70]. A study aimed at intervening in memory impairment by enhancing gut health found that oral administration of *s_Lactobacillus johnsonii* strains increased the abundance of *g_Lactobacillus* in the gut and decreased the gene copy number of *f_Enterobacteriaceae* to balance gut ecology, maintained the gut barrier by increasing the mRNA expression of tight junction proteins in the jejunum and ileum, and simultaneously downregulated the levels of TNF-*α* and hippocampal apoptosis and upregulated the expression level of BNDF, thereby reducing memory impairment [71]. Another experiment found that *s_Lactobacillus johnsonii* pretreatment inhibited the activation of the NLRP3 inflammasome and NF-*κ*B signalling in a *Salmonella infantis*-induced enteritis model [72]. These findings were confirmed in a *Salmonella typhimurium*-induced enteritis cell model. *s_Lactobacillus johnsonii* specifically inhibited the TLR4/NF-*κ*B/NLRP3 inflammatory signalling pathway, thereby downregulating the levels of inflammatory factors, such as IL-6, IL-1*β*, IL-18, and TNF-*α* [73]. In addition, studies of feeding *s_Lactobacillus johnsonii* to biologically bred diabetic-prone rats found that it can specifically mediate the self-cleavage of precursor Caspase-1 to mature Caspase-1 and reduce the expression levels of active Caspase-1 [74]. *s_Lactobacillus reuteri* showed a similar role in maintaining the gut mucosal barrier as *s_Lactobacillus_johnsonii* [75]. In addition, oral administration of *Lactobacillus reuteri* increased tryptophan metabolism and increased the level of the purine nucleoside adenosine, which can enhance tolerance to inflammatory stimuli [76], reduce the level of the inflammatory factor IL-1*β* [77], and upregulate the level of the immunosuppressive factor IL-10 [78]. *s_Lactobacillus_faecis* is a lactic acid producer, and studies on this bacterium are limited. However, *s_Lactobacillus_faecis* is closely related to *s_Lactobacillus murine* based on gene sequence phylogenetic analysis [79, 80], and *s_Lactobacillus murine* mediates the release of IL-10 by TLR2 receptors to restrict the inflammatory response [81]. Thus, *s_Lactobacillus_faecis* may represent a potential AD-modifying probiotic. *S_Helicobacter_rodentium* accounts for 78% of murine *H. pylori* infections, which can lead to inflammation and even death when animals have reduced immunity [82]. In contrast, human *H. pylori* can cause central nervous system damage and lead to AD [61]. There is no clear description related to *s_Ruminococcus_sp_N15.MGS-57*. However, *s_Ruminococcus gnavus*, which belong to the same genus, are strongly associated with inflammatory gut disease, and their metabolites can induce the production of TNF-*α*. Thus, *s_Ruminococcus_sp_N15.MGS-57* may represent a potential proinflammatory species [83].

JDYZF has a two-way effect on the gut microbiota structure of AD rats, reducing the abundance of some opportunistic pathogens while increasing the abundance of probiotics, which amplifies the effect of probiotics on the improvement of AD cognitive function. Through query and analysis, it was found that the functions of these altered species were related to obesity, diabetes, and inflammatory gut disease (Table 3). These findings indicated that JDYZF alleviated the background inflammation that led to the occurrence and progression of AD by regulating the gut microbiota.

In addition, *s_Lactobacillus_johnsonii* exhibits a specific inhibitory effect on inflammasome activation, as confirmed by immunohistochemical staining for Caspase-1. JDYZF reduced the abundance of some LPS-producing genera. LPS can circulate into the brain to activate multiple receptors. Caspase-11 can recognize LPS to initiate pyroptosis. Immunohistochemical staining for Caspase-11 also indirectly demonstrated a decrease in the amount of LPS circulating in the brain. These results are consistent with the previous results of this experiment [18], explaining the root cause of the inhibitory effect of JDYZF on pyroptosis-related proteins and inflammation levels.

## 5. Conclusion

JDYZF has a modulating effect on the gut microbiota of AD rats, which may be the basis for the cognitive protective effect of JDYZF. This result adds another step to the explanation of the mechanism of JDYZF and contributes to a potentially useful method for alternative AD therapy.

## Figures and Tables

**Table 1:** The body weight of the rats in each group after purchase and after gavage for one week and eight weeks.

W	*n*	After purchase	Gavage for 1 week	Gavage for 8 weeks
JDYZ.L	11	220.50 ± 6.51	357.00 ± 45.47^*∗*^	467.49 ± 27.41
JDYZ.M	11	220.51 ± 6.97	413.92 ± 32.57^##^	481.16 ± 38.61
JDYZ.H	11	219.34 ± 8.82	405.69 ± 42.40^##^	493.45 ± 47.64^#^
PG	10	219.61 ± 10.71	394.40 ± 38.30^##^	480.36 ± 43.00
MG	10	220.10 ± 8.07	399.18 ± 31.13	499.63 ± 36.39
CG	9	219.62 ± 7.93	338.76 ± 51.67^*∗∗*^	443.84 ± 54.30^*∗∗*^

Compared with the MG group, ^*∗*^*p* < 0.05 and ^*∗∗*^*p* < 0.01; compared with the CG group, ^#^*p* < 0.05 and ^##^*p* < 0.01.

**Table 2:** Raw PE, effective tags, and AvgLen of effective tags.

Group	*n*	Raw PE	Effective tags	AvgLen
CG	9	96538.22 ± 2923.08	60523.78 ± 2242.80	416.11 ± 1.83
MG	10	94723.80 ± 7665.92	58760.60 ± 3531.32	412.70 ± 0.82
PG	10	98760.30 ± 7004.07	60906.50 ± 3706.42	414.60 ± 1.27
JDYZ.H	11	97921.00 ± 6870.39	59599.82 ± 3515.13	414.82 ± 1.25
JDYZ.M	11	100802.73 ± 5701.94	62441.73 ± 3462.03	415.55 ± 1.37
JDYZ.L	11	104206.18 ± 4327.71	64347.36 ± 2607.63	416.55 ± 0.93

**Table 3:** Potential associations of some of the modulated microorganisms in this study with obesity, diabetes, inflammatory gut disease, and other diseases.

No.	Microorganisms	Obesity	Diabetes	Inflammatory gut disease	Other peripheral inflammation	Gut barrier and BBB damage	References
1	*p_Firmicutes*	↑	P↑	N	P↑	N	[32, 36, 84, 85]
2	*p_Bacteroidota*	↓	N	N	N	N	[36, 84]
3	*p_Actinobacteriota*	P↑	N	↓	P↓	↓	[36, 38, 84]
4	*p_Campilobacterota*	N	N	↑	↑	N	[40]
5	*g_lactobacillus*	P↓	↓	↓	↓	↓	[41, 86–89]
6	*g_prevotella*	P↑	C	P↓	C	↓	[45, 90–94]
7	*g_bacteroides*	↓	↓	P↓	P↓	P↓	[46, 47, 95–97]
8	*g_Christensenellaceae_R-7_group*	↓	P↓	↓	P↓	P↓	[48, 98–100]
9	*g_Rikenellaceae_RC9_gut_group*	↓	P↓	↓	P↓	P↓	[49, 50, 101, 102]
10	*g_Blautia*	↓	↓	↓	↓	↓	[51, 103]
11	*g_Lachnospiraceae-NK4A136-group*	P↑	P↑	P↑	P↑	P↑	[52–54]
12	*g_Anaerobiospirillum*	P↑	N	↑	↑	N	[55, 104]
13	*g_Turicibacter*	C	P↑	P↑	P↑	P↑	[56, 105–108]
14	*g_Oscillibacter*	C	C	↑	C	P↑	[57, 109–115]
15	*g_Desulfovibrio*	↑	P↑	P↑	↑	P↑	[60, 93, 116–118]
16	*g_Helicobacter*	↑	P↑	↑	↑	↑	[61, 119]
17	*g_Intestinimonas*	↑	P↑	P↑	C	↑	[63, 64, 120–122]
18	*g_Streptococcus*	↑	↑	↑	↑	↑	[66–69, 84]
19	*s_Lactobacillus_johnsonii*	↓	↓	↓	↓	↓	[71–74, 123]
20	*s_Lactobacillus_reuteri*	C	↓	↓	↓	↓	[75, 77, 124, 125]
21	*s_Lactobacillus_faecis*	N	N	P↓	P↓	P↓	[80, 81]
22	*s_Helicobacter_rodentium*	P↑	P↑	P↑	↑	P↑	[61, 82, 119]
23	*s_Ruminococcus_sp_N15.MGS-57*	P↑	P↑	P↑	P↑	C	[63, 83, 96, 126, 127]

Note: ↑/↓: positive/negative effects on the occurrence of disease; P↑/P↓: possibly positive/possibly negative effects on the occurrence of disease; N: not sure yet; C: controversial.

## Data Availability

The data used to support the results of this study are available from the corresponding author (M. L.).

## References

[1] Supnet C., Bezprozvanny I. (2011). Presenilins function in ER calcium leak and Alzheimer’s disease pathogenesis. *Cell Calcium*.

[2] Jiang J., Liu H., Wang Z. (2021). Electroacupuncture could balance the gut microbiota and improve the learning and memory abilities of Alzheimer’s disease animal model. *PLoS One*.

[3] Liu Q., Xi Y., Wang Q. (2021). Mannan oligosaccharide attenuates cognitive and behavioral disorders in the 5xFAD Alzheimer’s disease mouse model via regulating the gut microbiota-brain axis. *Brain, Behavior, and Immunity*.

[4] Alzheimer’s Association Report (2021). 2021 Alzheimer’s disease facts and figures. *Alzheimers Dement*.

[5] Martin T., Abushakra S., Hey J. A., Porsteinsson A., Sabbagh M. (2020). Aducanumab, gantenerumab, BAN2401, and ALZ-801-the first wave of amyloid-targeting drugs for Alzheimer’s disease with potential for near term approval. *Alzheimer’s Research & Therapy*.

[6] Knopman D. S., Jones D. T., Greicius M. D. (2021). Failure to demonstrate efficacy of aducanumab: an analysis of the EMERGE and ENGAGE trials as reported by Biogen, December 2019. *Alzheimer’s and Dementia*.

[7] Soscia S. J., Kirby J. E., Washicosky K. J. (2010). The Alzheimer’s disease-associated amyloid beta-protein is an antimicrobial peptide. *PLoS One*.

[8] Kumar D. K. V., Choi S. H., Washicosky K. J. (2016). Amyloid-*β* peptide protects against microbial infection in mouse and worm models of Alzheimer’s disease. *Science Translational Medicine*.

[9] Chacko A., Delbaz A., Walkden H. (2022). Chlamydia pneumoniae can infect the central nervous system via the olfactory and trigeminal nerves and contributes to Alzheimer’s disease risk. *Scientific Reports*.

[10] Megur A., Baltriukienė D., Bukelskienė V., Burokas A. (2020). The microbiota-gut-brain Axis and Alzheimer’s disease: neuroinflammation is to blame?. *Nutrients*.

[11] Wenzel T. J., Gates E. J., Ranger A. L., Klegeris A. (2020). Short-chain fatty acids (SCFAs) alone or in combination regulate select immune functions of microglia-like cells. *Molecular and Cellular Neuroscience*.

[12] Sun Y., Sommerville N. R., Liu J. Y. H. (2020). Intra-gastrointestinal amyloid-*β*1-42 oligomers perturb enteric function and induce Alzheimer’s disease pathology. *Journal of Physiology*.

[13] Sochocka M., Donskow-Łysoniewska K., Diniz B. S., Kurpas D., Brzozowska E., Leszek J. (2019). The gut microbiome alterations and inflammation-driven pathogenesis of Alzheimer’s disease-a critical review. *Molecular Neurobiology*.

[14] Evans J. M., Morris L. S., Marchesi J. R. (2013). The gut microbiome: the role of a virtual organ in the endocrinology of the host. *Journal of Endocrinology*.

[15] Lombardi V. C., De Meirleir K. L., Subramanian K. (2018). Nutritional modulation of the intestinal microbiota; future opportunities for the prevention and treatment of neuroimmune and neuroinflammatory disease. *The Journal of Nutritional Biochemistry*.

[16] Goyal D., Ali S. A., Singh R. K. (2021). Emerging role of gut microbiota in modulation of neuroinflammation and neurodegeneration with emphasis on Alzheimer’s disease. *Progress in Neuro-Psychopharmacology and Biological Psychiatry*.

[17] Minamisawa M., Sato Y., Ishiguro E. (2021). Amelioration of Alzheimer’s disease by gut-pancreas-liver-brain interaction in an App knock-in mouse model. *Life*.

[18] Wang J., Zhu X., Li Y., Zhang P., Wang T., Li M. (2022). Jiedu-yizhi formula improves cognitive impairment in an A *β* 25-35-induced rat model of Alzheimer’s disease by inhibiting pyroptosis. *Evidence-based Complementary and Alternative Medicine*.

[19] Liu Y., Wei M., Yue K. (2018). Study on urine metabolic profile of a*β*25–35-induced Alzheimer’s disease using UHPLC-Q-TOF-MS. *Neuroscience*.

[20] Zuo H., Liu X., Wang D. (2018). RKIP-Mediated NF-*κ*B Signaling is involved in ELF-MF-mediated improvement in AD rat. *International Journal of Medical Sciences*.

[21] Magoč T., Salzberg S. L. (2011). FLASH: fast length adjustment of short reads to improve genome assemblies. *Bioinformatics*.

[22] Caporaso J. G., Kuczynski J., Stombaugh J. (2010). QIIME allows analysis of high-throughput community sequencing data. *Nature Methods*.

[23] Edgar R. C., Haas B. J., Clemente J. C., Quince C., Knight R. (2011). UCHIME improves sensitivity and speed of chimera detection. *Bioinformatics*.

[24] Haas B. J., Gevers D., Earl A. M. (2011). Chimeric 16S rRNA sequence formation and detection in Sanger and 454-pyrosequenced PCR amplicons. *Genome Research*.

[25] Edgar R. C. (2013). UPARSE: highly accurate OTU sequences from microbial amplicon reads. *Nature Methods*.

[26] Quast C., Pruesse E., Yilmaz P. (2012). The SILVA ribosomal RNA gene database project: improved data processing and web-based tools. *Nucleic Acids Research*.

[27] Ramos-Vara J. A. (2017). Principles and methods of immunohistochemistry. *Methods in Molecular Biology*.

[28] Vogt N. M., Kerby R. L., Dill-McFarland K. A. (2017). Gut microbiome alterations in Alzheimer’s disease. *Scientific Reports*.

[29] Wang X., Sun G., Feng T. (2019). Sodium oligomannate therapeutically remodels gut microbiota and suppresses gut bacterial amino acids-shaped neuroinflammation to inhibit Alzheimer’s disease progression. *Cell Research*.

[30] Lee H. J., Lee K. E., Kim J. K., Kim D. H. (2019). Suppression of gut dysbiosis by Bifidobacterium longum alleviates cognitive decline in 5XFAD transgenic and aged mice. *Scientific Reports*.

[31] Da Silva C. C., Monteil M. A., Davis E. M. (2020). Overweight and obesity in children are associated with an abundance of firmicutes and reduction of bifidobacterium in their gastrointestinal microbiota. *Childhood Obesity*.

[32] Orbe-Orihuela Y. C., Lagunas-Martínez A., Bahena-Román M. (2017). High relative abundance of firmicutes and increased TNF-*α* levels correlate with obesity in children. *Salud Publica de Mexico*.

[33] Killingsworth J., Sawmiller D., Shytle R. D. (2020). Propionate and Alzheimer’s disease. *Frontiers in Aging Neuroscience*.

[34] Zhao Y., Lukiw W. J. (2018). Bacteroidetes neurotoxins and inflammatory neurodegeneration. *Molecular Neurobiology*.

[35] Liu M., Hu R., Guo Y. (2020). Influence of Lactobacillus reuteri SL001 on intestinal microbiota in AD model mice and C57BL/6 mice. *Sheng Wu Gong Cheng Xue Bao*.

[36] Riva A., Borgo F., Lassandro C. (2017). Pediatric obesity is associated with an altered gut microbiota and discordant shifts in firmicutes populations. *Environmental Microbiology*.

[37] Hardy H., Harris J., Lyon E., Beal J., Foey A. (2013). Probiotics, prebiotics and immunomodulation of gut mucosal defences: homeostasis and immunopathology. *Nutrients*.

[38] Scarpellini E., Tack J. (2012). Obesity and metabolic syndrome: an inflammatory condition. *Digestive Diseases*.

[39] Groeger D., O’Mahony L., Murphy E. F. (2013). *Bifidobacterium infantis* 35624 modulates host inflammatory processes beyond the gut. *Gut Microbes*.

[40] Fitzgerald C. (2015). Campylobacter. *Clinics in Laboratory Medicine*.

[41] Ivakhniuk T., Yu I. (2021). Intestinal microbiota in alzheimer’s disease. *Georgian Medical News*.

[42] Nimgampalle M., Kuna Y. (2017). Anti-Alzheimer properties of probiotic, lactobacillus plantarum MTCC 1325 in Alzheimer’s disease induced albino rats. *Journal of Clinical and Diagnostic Research*.

[43] Shamsipour S., Sharifi G., Taghian F. (2021). An 8-week administration of bifidobacterium bifidum and lactobacillus plantarum combined with exercise training alleviates neurotoxicity of A*β* and spatial learning via acetylcholine in alzheimer rat model. *Journal of Molecular Neuroscience*.

[44] Ni Y., Yang X., Zheng L. (2019). Lactobacillus and bifidobacterium improves physiological function and cognitive ability in aged mice by the regulation of gut microbiota. *Molecular Nutrition & Food Research*.

[45] Prykhodko O., Sandberg J., Burleigh S., Bjorck I., Nilsson A., Fak Hallenius F. (2018). Impact of rye kernel-based evening meal on microbiota composition of young healthy lean volunteers with an emphasis on their hormonal and appetite regulations, and blood levels of brain-derived neurotrophic factor. *Frontiers in Nutrition*.

[46] Tan H., Zhai Q., Chen W. (2019). Investigations of Bacteroides spp. towards next-generation probiotics. *Food Research International*.

[47] Tamana S. K., Tun H. M., Konya T. (2021). Bacteroides-dominant gut microbiome of late infancy is associated with enhanced neurodevelopment. *Gut Microbes*.

[48] Waters J. L., Ley R. E. (2019). The human gut bacteria Christensenellaceae are widespread, heritable, and associated with health. *BMC Biology*.

[49] Tavella T., Rampelli S., Guidarelli G. (2021). Elevated gut microbiome abundance of Christensenellaceae, Porphyromonadaceae and Rikenellaceae is associated with reduced visceral adipose tissue and healthier metabolic profile in Italian elderly. *Gut Microbes*.

[50] Bian X., Wu W., Yang L. (2019). Administration of akkermansia muciniphila ameliorates dextran sulfate sodium-induced ulcerative colitis in mice. *Frontiers in Microbiology*.

[51] Liu X., Mao B., Gu J. (2021). Blautia-a new functional genus with potential probiotic properties?. *Gut Microbes*.

[52] Vacca M., Celano G., Calabrese F. M., Portincasa P., Gobbetti M., De Angelis M. (2020). The controversial role of human gut Lachnospiraceae. *Microorganisms*.

[53] Wang P., Gao J., Ke W. (2020). Resveratrol reduces obesity in high-fat diet-fed mice via modulating the composition and metabolic function of the gut microbiota. *Free Radical Biology and Medicine*.

[54] Wang P., Li D., Ke W., Liang D., Hu X., Chen F. (2020). Resveratrol-induced gut microbiota reduces obesity in high-fat diet-fed mice. *International Journal of Obesity*.

[55] Alguacil-Guillen M., Ramos-Ruperto L., Ramos Ramos J. C. (2019). MALDI-TOF MS for rapid diagnosis of Anaerobiospirillum succiniciproducens, an unusual causative agent of bacteraemia in humans. Two case reports and literature review. *Anaerobe*.

[56] Clark A., Mach N (2016). Exercise-induced stress behavior, gut-microbiota-brain axis and diet: a systematic review for athletes. *Journal of the International Society of Sports Nutrition*.

[57] Wang C.-S.-E., Li W.-B., Wang H.-Y. (2018). VSL#3 can prevent ulcerative colitis-associated carcinogenesis in mice. *World Journal of Gastroenterology*.

[58] Barandouzi Z. A., Starkweather A. R., Henderson W. A., Gyamfi A., Cong X. S. (2020). Altered composition of gut microbiota in depression: a systematic review. *Frontiers in Psychiatry*.

[59] Murros K. E., Huynh Vy A., Takala T. M., Saris P. E. J. (2021). Desulfovibrio bacteria are associated with Parkinson’s disease. *Frontiers in Cellular and Infection Microbiology*.

[60] Simpson C. A., Diaz-Arteche C., Eliby D., Schwartz O. S., Simmons J. G., Cowan C. S. (2021). The gut microbiota in anxiety and depression - a systematic review. *Clinical Psychology Review*.

[61] Doulberis M., Kotronis G., Thomann R. (2018). Impact of *Helicobacter pylori* on Alzheimer’s disease: what do we know so far?. *Helicobacter*.

[62] Contaldi F., Capuano F., Fulgione A. (2017). The hypothesis that *Helicobacter pylori* predisposes to Alzheimer’s disease is biologically plausible. *Scientific Reports*.

[63] Lin H., An Y., Tang H., Wang Y. (2019). Alterations of bile acids and gut microbiota in obesity induced by high fat diet in rat model. *Journal of Agricultural and Food Chemistry*.

[64] Du G., Dong W., Yang Q. (2020). Altered gut microbiota related to inflammatory responses in patients with Huntington’s disease. *Frontiers in Immunology*.

[65] Li N., Wang J., Liu P., Li J., Xu C. (2022). Multi-omics reveals that Bifidobacterium breve M-16V may alleviate the immune dysregulation caused by nanopolystyrene. *Environment International*.

[66] Venkatesh K. K., Vladutiu C. J., Strauss R. A. (2020). Association between maternal obesity and group B Streptococcus colonization in a national U.S. Cohort. *Journal of Women’s Health*.

[67] Mallick B., Nath P., Praharaj D. L., Panigrahi S. C., Anand A. (2020). Streptococcus agalactiae-related splenic abscess in uncontrolled diabetes mellitus. *Cureus*.

[68] Little R., Wine E., Kamath B. M., Griffiths A. M., Ricciuto A. (2020). Gut microbiome in primary sclerosing cholangitis: a review. *World Journal of Gastroenterology*.

[69] Reinscheid F. (2021). A new proposal for the causative agent of the sporadic form of Alzheimer’s disease. *Medical Hypotheses*.

[70] He T., Zhu Y.-H., Yu J. (2019). Lactobacillus johnsonii L531 reduces pathogen load and helps maintain short-chain fatty acid levels in the intestines of pigs challenged with Sal monella enterica Infantis. *Veterinary Microbiology*.

[71] Wang H., Sun Y., Xin J. (2020). Lactobacillus johnsonii BS15 prevents psychological stress-induced memory dysfunction in mice by modulating the gut-brain Axis. *Frontiers in Microbiology*.

[72] Xia B., Yu J., He T. (2020). Lactobacillus johnsonii L531 ameliorates enteritis via elimination of damaged mitochondria and suppression of SQSTM1-dependent mitophagy in a Salmonella infantis model of piglet diarrhea. *The FASEB Journal*.

[73] Chen S., Li Y., Chu B. (2021). Lactobacillus johnsonii L531 alleviates the damage caused by Salmonella typhimurium via inhibiting TLR4, NF-*κ*B, and NLRP3 inflammasome signaling pathways. *Microorganisms*.

[74] Teixeira L. D., Kling D. N., Lorca G. L., Gonzalez C. (2018). Lactobacillus johnsonii N6.2 diminishes caspase-1 maturation in the gastrointestinal system of diabetes prone rats. *Beneficial Microbes*.

[75] Yi H., Wang L., Xiong Y. (2018). Lactobacillus reuteri LR1 improved expression of genes of tight junction proteins via the MLCK pathway in IPEC-1 cells during infection with enterotoxigenic *Escherichia coli* K88. *Mediators of Inflammation*.

[76] Liu Y., Tian X., He B. (2019). Lactobacillus reuteri DSM 17938 feeding of healthy newborn mice regulates immune responses while modulating gut microbiota and boosting beneficial metabolites. *American Journal of Physiology - Gastrointestinal and Liver Physiology*.

[77] Hsieh M.-C., Tsai W.-H., Jheng Yu-P. (2018). The beneficial effects of Lactobacillus reuteri ADR-1 or ADR-3 consumption on type 2 diabetes mellitus: a randomized, double-blinded, placebo-controlled trial. *Scientific Reports*.

[78] Engevik M. A., Ruan W., Esparza M. (2021). Immunomodulation of dendritic cells by Lactobacillus reuteri surface components and metabolites. *Physics Reports*.

[79] Endo A., Irisawa T., Futagawa-Endo Y., Salminen S., Ohkuma M., Dicks L. (2013). Lactobacillus faecis sp. nov., isolated from animal faeces. *International Journal of Systematic and Evolutionary Microbiology*.

[80] Yang M., Meng F., Gu W. (2021). Influence of polysaccharides from polygonatum kingianum on short-chain fatty acid production and quorum sensing in lactobacillus faecis. *Frontiers in Microbiology*.

[81] Hu J., Deng F., Zhao B. (2022). Lactobacillus murinus alleviate intestinal ischemia/reperfusion injury through promoting the release of interleukin-10 from M2 macrophages via Toll-like receptor 2 signaling. *Microbiome*.

[82] Wasimuddin, Čížková D., Bryja J., Albrechtova J., Hauffe H. C., Pialek J. (2012). High prevalence and species diversity of Helicobacter spp. detected in wild house mice. *Applied and Environmental Microbiology*.

[83] Henke M. T., Kenny D. J., Cassilly C. D., Vlamakis H., Xavier R. J., Clardy J. (2019). Ruminococcus gnavus, a member of the human gut microbiome associated with Crohn’s disease, produces an inflammatory polysaccharide. *Proceedings of the National Academy of Sciences of the United States of America*.

[84] Singer-Englar T., Barlow G., Mathur R. (2019). Obesity, diabetes, and the gut microbiome: an updated review. *Expert Review of Gastroenterology & Hepatology*.

[85] Umirah F., Neoh C. F., Ramasamy K., Lim S. M. (2021). Differential gut microbiota composition between type 2 diabetes mellitus patients and healthy controls: a systematic review. *Diabetes Research and Clinical Practice*.

[86] Sergeev I. N., Aljutaily T., Walton G., Huarte E. (2020). Effects of synbiotic supplement on human gut microbiota, body composition and weight loss in obesity. *Nutrients*.

[87] Yan F., Li N., Shi J. (2019). Lactobacillus acidophilus alleviates type 2 diabetes by regulating hepatic glucose, lipid metabolism and gut microbiota in mice. *Food & Function*.

[88] Wilck N., Matus M. G., Kearney S. M. (2017). Salt-responsive gut commensal modulates T H 17 axis and disease. *Nature*.

[89] Yang X., Yu D., Xue L., Li H., Du J. (2020). Probiotics modulate the microbiota-gut-brain axis and improve memory deficits in aged SAMP8 mice. *Acta Pharmaceutica Sinica B*.

[90] Stanislawski M. A., Dabelea D., Lange L. A., Wagner B. D., Lozupone C. A. (2019). Gut microbiota phenotypes of obesity. *NPJ Biofilms Microbiomes*.

[91] Medina-Vera I., Sanchez-Tapia M., Noriega-López L. (2019). A dietary intervention with functional foods reduces metabolic endotoxaemia and attenuates biochemical abnormalities by modifying faecal microbiota in people with type 2 diabetes. *Diabetes & Metabolism*.

[92] Asnicar F., Berry S. E., Valdes A. M. (2021). Microbiome connections with host metabolism and habitual diet from 1, 098 deeply phenotyped individuals. *Nature Medicine*.

[93] Liu B., Piao X., Niu W. (2020). Kuijieyuan decoction improved intestinal barrier injury of ulcerative colitis by affecting TLR4-dependent PI3K/AKT/NF-*κ*B oxidative and inflammatory signaling and gut microbiota. *Frontiers in Pharmacology*.

[94] Larsen J. M. (2017). The immune response to Prevotella bacteria in chronic inflammatory disease. *Immunology*.

[95] Amabebe E., Robert F. O., Agbalalah T., Orubu E. S. F. (2020). Microbial dysbiosis-induced obesity: role of gut microbiota in homoeostasis of energy metabolism. *British Journal of Nutrition*.

[96] Gurung M., Li Z., You H. (2020). Role of gut microbiota in type 2 diabetes pathophysiology. *EBioMedicine*.

[97] Zhou F., Li Yi-L., Zhang X. (2021). Polyphenols from fu brick tea reduce obesity via modulation of gut microbiota and gut microbiota-related intestinal oxidative stress and barrier function. *Journal of Agricultural and Food Chemistry*.

[98] Chen Z., Radjabzadeh D., Chen L. (2021). Association of insulin resistance and type 2 diabetes with gut microbial diversity: a microbiome-wide analysis from population studies. *JAMA Network Open*.

[99] Coello K., Hansen T. H., Sørensen N. (2021). Affective disorders impact prevalence of Flavonifractor and abundance of Christensenellaceae in gut microbiota. *Progress in Neuro-Psychopharmacology and Biological Psychiatry*.

[100] Jing Y., Yu Y., Bai F. (2021). Effect of fecal microbiota transplantation on neurological restoration in a spinal cord injury mouse model: involvement of brain-gut axis. *Microbiome*.

[101] Miao Z., Lin J.-S., Mao Y. (2020). Erythrocyte n-6 polyunsaturated fatty acids, gut microbiota, and incident type 2 diabetes: a prospective cohort study. *Diabetes Care*.

[102] Tan W., Zhang Q., Dong Z. (2020). Phosphatidylcholine ameliorates LPS-induced systemic inflammation and cognitive impairments via mediating the gut-brain Axis balance. *Journal of Agricultural and Food Chemistry*.

[103] Liu C., Cheng Y., Guo Y., Qian H. (2021). Magnesium-L-threonate alleviate colonic inflammation and memory impairment in chronic-plus-binge alcohol feeding mice. *Brain Research Bulletin*.

[104] Li Y., Rahman S. U., Huang Y. (2020). Green tea polyphenols decrease weight gain, ameliorate alteration of gut microbiota, and mitigate intestinal inflammation in canines with high-fat-diet-induced obesity. *The Journal of Nutritional Biochemistry*.

[105] Li T.-T., Huang Zi-R., Jia R.-Bo, Lv X. C., Zhao C., Liu B. (2021). Spirulina platensis polysaccharides attenuate lipid and carbohydrate metabolism disorder in high-sucrose and high-fat diet-fed rats in association with intestinal microbiota. *Food Research International*.

[106] Zhao Q., Hou D., Fu Y., Xue Y., Guan X., Shen Q. (2021). Adzuki bean alleviates obesity and insulin resistance induced by a high-fat diet and modulates gut microbiota in mice. *Nutrients*.

[107] Horie M., Miura T., Hirakata S. (2017). Comparative analysis of the intestinal flora in type 2 diabetes and nondiabetic mice. *Experimental Animals*.

[108] Wang S., Chen H., Wen X. (2021). The efficacy of fecal microbiota transplantation in experimental autoimmune encephalomyelitis: transcriptome and gut microbiota profiling. *Journal of Immunology Research*.

[109] Kong C., Gao R., Yan X., Huang L., Qin H. (2019). Probiotics improve gut microbiota dysbiosis in obese mice fed a high-fat or high-sucrose diet. *Nutrition*.

[110] Yuan G., Tan M., Chen X. (2021). Punicic acid ameliorates obesity and liver steatosis by regulating gut microbiota composition in mice. *Food & Function*.

[111] Song Y., Wu M.-S., Tao G., Lu M., Lin J., Huang J. (2020). Feruloylated oligosaccharides and ferulic acid alter gut microbiome to alleviate diabetic syndrome. *Food Research International*.

[112] Hu R., Zeng F., Wu L. (2019). Fermented carrot juice attenuates type 2 diabetes by mediating gut microbiota in rats. *Food & Function*.

[113] Zhang Y., Chen L., Hu M. (2020). Dietary type 2 resistant starch improves systemic inflammation and intestinal permeability by modulating microbiota and metabolites in aged mice on high-fat diet. *Aging (Albany NY)*.

[114] Hsiao Yu-P., Chen H.-L., Tsai J.-N. (2021). Administration of lactobacillus reuteri combined with Clostridium butyricum attenuates cisplatin-induced renal damage by gut microbiota reconstitution, increasing butyric acid production, and suppressing renal inflammation. *Nutrients*.

[115] Huang P., Jiang A., Wang X. (2021). NMN maintains intestinal homeostasis by regulating the gut microbiota. *Frontiers in Nutrition*.

[116] Petersen C., Bell R., Klag K. A. (2019). T cell-mediated regulation of the microbiota protects against obesity. *Science*.

[117] Zhang P.-P., Li L.-L., Han X. (2020). Fecal microbiota transplantation improves metabolism and gut microbiome composition in db/db mice. *Acta Pharmacologica Sinica*.

[118] Cui H., Cai Y., Wang Li (2018). Berberine regulates treg/Th17 balance to treat ulcerative colitis through modulating the gut microbiota in the colon. *Frontiers in Pharmacology*.

[119] Furuto Y., Kawamura M., Yamashita J. (2021). Relationship between *Helicobacter pylori* infection and arteriosclerosis. *International Journal of General Medicine*.

[120] Van Buiten C. B., Wu G., Lam Y. Y., Zhao L., Raskin I. (2021). Elemental iron modifies the redox environment of the gastrointestinal tract: a novel therapeutic target and test for metabolic syndrome. *Free Radical Biology and Medicine*.

[121] Wu Y., Chen Y., Li Q. (2021). Tetrahydrocurcumin alleviates allergic airway inflammation in asthmatic mice by modulating the gut microbiota. *Food & Function*.

[122] Geng S., Yang L., Cheng F. (2019). Gut microbiota are associated with psychological stress-induced defections in intestinal and blood-brain barriers. *Frontiers in Microbiology*.

[123] Yang G., Hong E., Oh S., Kim E. (2020). Non-viable lactobacillus johnsonii JNU3402 protects against diet-induced obesity. *Foods*.

[124] Oh J.-H., Schueler K. L., Stapleton D. S. (2020). Secretion of recombinant interleukin-22 by engineered lactobacillus reuteri reduces fatty liver disease in a mouse model of diet-induced obesity. *mSphere*.

[125] Crovesy L., Masterson D., Rosado E. L. (2020). Profile of the gut microbiota of adults with obesity: a systematic review. *European Journal of Clinical Nutrition*.

[126] Qi L., Mao H., Lu X., Shi T., Wang J. (2021). Cinnamaldehyde promotes the intestinal barrier functions and reshapes gut microbiome in early weaned rats. *Frontiers in Nutrition*.

[127] Yang S., Hu T., Liu H. (2021). Akebia saponin D ameliorates metabolic syndrome (MetS) via remodeling gut microbiota and attenuating intestinal barrier injury. *Biomedicine & Pharmacotherapy*.

